# Plasticity in Hydraulic Architecture: Riparian Trees Respond to Increased Temperatures With Genotype‐Specific Adjustments to Leaf Traits

**DOI:** 10.1002/ece3.70683

**Published:** 2024-12-12

**Authors:** Iris J. Garthwaite, Catherine Lepp, Zyled S. R. Maldonado, Davis Blasini, Kevin C. Grady, Catherine A. Gehring, Kevin R. Hultine, Thomas G. Whitham, Gerard J. Allan, Rebecca J. Best

**Affiliations:** ^1^ School of Earth and Sustainability Northern Arizona University Flagstaff Arizona USA; ^2^ School of Life Sciences Arizona State University Tempe Arizona USA; ^3^ School of Forestry Northern Arizona University Flagstaff Arizona USA; ^4^ Department of Biological Science Northern Arizona University Flagstaff Arizona USA; ^5^ Center for Adaptable Western Landscapes Northern Arizona University Flagstaff Arizona USA; ^6^ Department of Research, Conservation and Collections Desert Botanical Garden Phoenix Arizona USA

**Keywords:** experimental common garden, phenotypic plasticity, stomatal density, vein density, venation network

## Abstract

Climate means and variability are shifting rapidly, leading to mismatches between climate and locally adapted plant traits. Phenotypic plasticity, the ability of a plant to respond to environmental conditions within a lifetime, may provide a buffer for plants to persist under increasing temperature and water stress. We used two reciprocal common gardens across a steep temperature gradient to investigate plasticity in six populations of Fremont cottonwood, an important foundation tree species in arid riparian ecosystems. We investigated two components of leaf hydraulic architecture: Leaf venation and stomatal morphology, both of which regulate leaf water potential and photosynthesis. These traits will likely affect plant performance under climate stressors, but it is unclear whether they are controlled by genetic or environmental factors and whether they respond to the environment in parallel or independent directions. We found that: (1) Populations had divergent responses to a hotter growing environment, increasing or decreasing vein density. (2) Populations showed surprisingly independent responses of venation vs. stomatal traits. (3) As a result of these different responses, plasticity in hydraulic architecture traits was not predictable from historic climate conditions at population source locations and often varied substantially within populations. (4) Hydraulic architecture was clearly linked to growth, with higher vein and stomatal density predicting greater tree growth in the hottest growing environment. However, higher plasticity in these traits did not increase average growth across multiple environments. Thus, *P. fremontii* populations and genotypes vary in their capacity to adjust their leaf hydraulic architecture and support growth in contrasting environments, but this plasticity is not clearly predictable or beneficial. Identifying genotypes suitable for future conditions will depend on the relative importance of multiple traits and on both evolutionary and ecological responses to changing temperature and water availability.

## Abstract 2

We used reciprocal common gardens across a steep climate gradient in the Southwestern USA to investigate how past climate conditions might explain the evolution of population differences in leaf hydraulic traits. We found that genetically divergent populations differ in their leaf venation and stomatal traits and in their trait plasticities. Population trait means predicted tree growth in hot conditions, whereas the evolutionary origins and ecological consequences of population variation in plasticity were much more complex.

## Graphical abstract

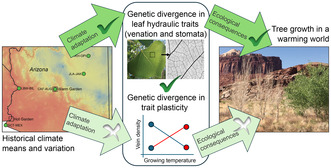

## 1 Introduction

Climate gradients across the range of many widely distributed plant species have led to the evolution of geographic clines or ecotypes, resulting in high levels of intraspecific phenotypic divergence (Aitken and Whitlock 2013). To understand and predict the way these species will respond to climate change, we must disentangle the genetic and environmental factors that constrain functional traits and their performance (Nicotra et al. 2010). Because of the increased probability of water limitation under drought conditions intensified by climate change (Cook et al. 2022), it is particularly important to quantify genetic and environmental drivers of variation in traits related to water use efficiency and hydraulic conductance (Challis et al. 2022). For example, traits such as stomatal density can vary across genotypes or populations (Abrams and Kubiske 1990) and can also respond to growing conditions (Abrams 1988). Quantifying these components of trait variation and understanding how they respond to environmental change will be critical for planning restoration and conservation efforts across a species range.

Phenotypic plasticity is a mechanism by which plants can alter their traits to respond to environmental conditions. This is particularly important for long‐lived trees species, which are subject to a range of climate conditions during their life span (Nicotra et al. 2010). Phenotypic plasticity is widespread in plant species, but individual genotypes within a species can vary in the magnitude and direction of plasticity due to differences in the environmental conditions to which they have adapted (Franks, Weber, and Aitken 2014). Specifically, phenotypic plasticity is predicted to evolve in places with more predictable environmental or climate variation (Botero et al. 2015; Leung et al. 2020). In the riparian tree *Populus fremontii* , S. Wats. (Fremont cottonwood), populations from hotter climates exhibit up to four times greater plasticity in phenological traits (timing of bud flush and bud set) than cold‐adapted northern populations (Cooper et al. 2019). Because of their wide climatic range, genetic variation leading to local adaptation, and differential evolution of plasticity (Cooper et al. 2022), tree species like *P. fremontii* are likely to have complex genetic and environmental determinants of their venation and stomatal traits. However, few studies have attempted to determine the distribution and evolutionary origins of variation in these types of tree leaf traits at the landscape scale.

**FIGURE 1:**
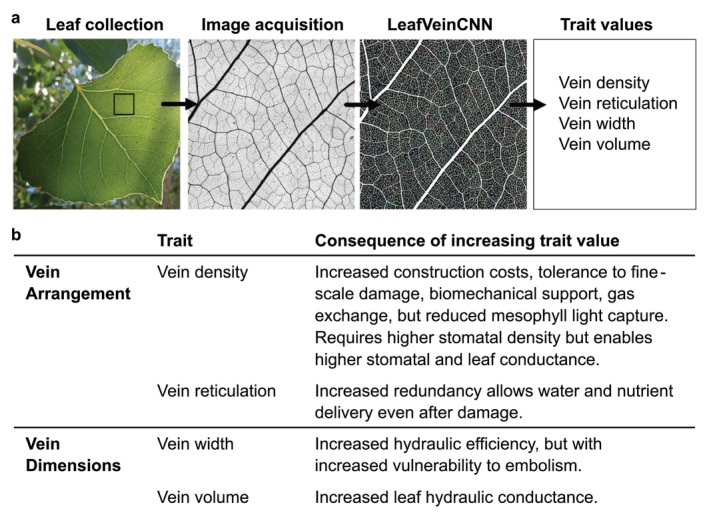
Leaf venation traits used in this study, from (a) extraction and quantification to (b) interpretation. Consequences of changing venation values are based on Sack and Scoffoni (2013).

Leaf hydraulic architecture traits are a key determinant of plant performance (Sack et al. 2012; Sack and Scoffoni 2013) and are particularly important to understand in ecosystems responding to thermal stress and/or water limitation. Leaf venation plays a major role in leaf hydraulic conductance, which determines how efficiently water is transported within a leaf and influences the movement of water throughout the whole plant vascular system (Brodribb and Holbrook 2004). The architecture of these “superhighways” varies widely across species and is thought to be a key innovation in the evolution of angiosperms (Boyce et al. 2009; de Boer et al. 2012; Sack and Scoffoni 2013). Most angiosperms (all dicots and some monocots) have a netted venation network (Figure 1a) with a hierarchy of vein orders that efficiently transport water, hormones, and nutrients throughout the leaf lamina (Sack and Scoffoni 2013). These veins lead to stomata, small pores on the surfaces of leaves, which also play a key role in regulating leaf hydraulic functioning and gas exchange (Figure 1b). While leaf veins provide pathways for water transport in leaves, stomatal density (number of stomata per unit of leaf area) and stomatal size can even more directly regulate whole plant water loss and carbon uptake. Stomatal density and size are usually negatively correlated and combine to govern maximum stomatal conductance (Milla, de Diego‐Vico, and Martín‐Robles 2013). Both venation traits (Read and Stokes 2006; Scoffoni et al. 2011; Vincent 1990; Sack et al. 2008) and stomatal traits (Casson and Gray 2008; Miyazawa, Livingston, and Turpin 2006) vary among and within species, but we know much less about the extent and drivers of intraspecific variation (Pritzkow et al. 2020).

The morphology and spatial arrangement of leaf veins and stomata are expected to be linked in plants to maximize function (Brodribb, McAdam, and Carins Murphy 2017). Coordination between these water delivery and water loss systems (Brodribb, McAdam, and Carins Murphy 2017) can help plants to balance water supply and demand (Brodribb and Jordan 2011; Sack and Scoffoni 2013), photosynthesis, and temperature regulation (Blackman, Brodribb, and Jordan 2009; Brodribb and Holbrook 2007). For example, plants often respond to low soil water availability and/or high evaporative demand by closing their stomata in order to avoid steep reductions in leaf water potentials (Brodribb and Holbrook 2003), but this limits both photosynthesis and leaf temperature regulation. Thus, the tradeoff between photosynthetic capacity, water retention, and cooling is a key component of adaptation to heat and water stress (Blasini et al. 2021, 2022; Hultine et al. 2020a). To optimize this tradeoff, plants have evolved morphological strategies that allow them to operate near their tolerance thresholds (Brodribb and Holbrook 2004) and reduce vulnerability to temporary drought conditions (Nardini et al. 2014). For example, a high density of narrow veins with reticulate architecture can better withstand embolism and avoid hydraulic disruption (Blackman, Brodribb, and Jordan 2010; Sack et al. 2008). Additionally, minor veins can stay fully hydrated during hydraulic disruptions of larger veins, acting as water storage pools to aid in recovery and drought tolerance (Muries et al. 2019). Stomatal density can also affect key plant physiological traits such as water use efficiency and hydraulic efficiency (Bertolino, Caine, and Gray 2019). Plants can respond quickly to altered water potential gradients by closing their stomata, but the specific dimensions and spatial arrangement of leaf anatomical phenotypes constrain the efficiency and speed of these responses and are fixed within the leaf. For example, smaller stomata can open and close more quickly, responding efficiently to changing conditions (Hetherington and Woodward 2003). These key morphological traits can only be adjusted via plastic responses to growing conditions during annual leaf production or via long‐term adaptation to local conditions.

Although past studies have shown strong correlations between environmental variables and leaf hydraulic architecture traits (Blonder and Enquist 2014; Critchfield 1960; Liu et al. 2018; Uhl and Mosbrugger 1999), very little is known about how leaf hydraulic architecture traits are jointly determined by genetics and the environment. Both across and within species, higher venation density and higher stomatal density have often evolved in hot, arid climates with high potential evapotranspiration (Blonder et al. 2017; Liu et al. 2018; Sack and Scoffoni 2013). A recent study of *Populus fremontii* in a single common garden found that genotypes from hot environments had 67% higher stomatal density but 37% smaller stomatal size than genotypes from cooler environments (Blasini et al. 2021). However, plastic responses to environmental variation across space and time may modify these and other hydraulic trait differences among populations (Blackman et al. 2017; Challis et al. 2022). In this study, we take a genotype by environment approach to quantify these sources of variation in leaf hydraulic architecture traits for this important riparian foundation species across its southwestern climatic gradient. Understanding whether leaf hydraulic architecture traits are consistently different among locally adapted genotypes or are plastic in response to climate will help predict how trees from different source populations will respond to climate change.

As the dominant riparian tree species in the Southwestern USA, *Populus fremontii* is distributed across a broad climate gradient, ranging from the southern Sonoran Desert and the central California Valley to the upper reaches of the Colorado Plateau in northern Arizona and Utah (Ikeda et al. 2017). Populations of this species are locally adapted to very different climates, which has led to the identification of different ecotypes (Blasini et al. 2021; Cooper et al. 2019; Ikeda et al. 2017) with a mosaic of traits expressed across the species range (Blasini et al. 2021, 2022; Cooper et al. 2019; Cushman et al. 2014; Grady et al. 2013). Past studies indicate high levels of genetic differentiation in *P. fremontii* traits that have consequences for entire communities and ecosystem processes (LeRoy et al. 2007; Whitham et al. 2008). However, the traits of a particular genotype or population may also be stable or plastic in response to environmental signals. Recent studies have shown that the magnitude and direction of phenotypic plasticity in phenology (Cooper et al. 2019) and chemical defense (Eisenring et al. 2022) vary across populations and are under selection (Cooper et al. 2022). However, it remains unclear whether populations have evolved coordinated plastic responses across many traits, including those conferring tolerance of thermal stress and water limitation.

Our approach disentangles the effects of environmental and genetic factors to better predict how *Populus fremontii* will respond to environmental conditions and how responses may vary among genotypes and populations. We used two experimental common gardens reciprocally planted with multiple genotypes from each of six populations from across the climatic range of *P. fremontii* . Specifically, we tested four hypotheses. (1) Due to the wide distribution of *P. fremontii* populations across a steep climate gradient and the known genetic variation in *P. fremontii* functional traits, there will be significant variation in leaf hydraulic architecture traits and their plastic responses to growing conditions across populations and genotypes. Due to a history of selection by the stresses of high evapotranspiration, we expect populations from hotter locations to have higher average vein and stomatal densities. (2) Because both leaf venation and stomatal traits govern plant water use in series, changes in venation will be correlated with changes in stomata. (3) Further, plasticity in these multiple traits will be predictable from source population site climate variables due to local adaptation to climate fluctuations and extremes. In particular, we expect higher plasticity in populations with a history of predictable climate variability. (4) Finally, different leaf hydraulic architecture traits will be beneficial in different environments. Thus, trait values within an environment should predict local growth, and plasticity across environments should enable greater average growth rates across those environments.

Understanding plant responses to environmental signals is important for predicting whole plant performance under climate change, which continues to disproportionately impact riparian ecosystems in the Southwest (Brusca et al. 2013; Garfin et al. 2013). Increasing temperatures and changing precipitation patterns are already altering flow regimes, soil moisture, and plant water use under thermal stress (Garfin et al. 2013). Although it once formed gallery forests that dominated southwestern riparian corridors (Stromberg 1993), *P. fremontii* can experience up to 97% mortality in a single stand and 20.7% on average regionally (Gitlin et al. 2006). If plasticity in leaf hydraulic architecture varies among *P. fremontii* genotypes and populations, this may be a major determinant in which trees in which locations can survive future conditions. Understanding this important intraspecific variation in environmental responses could therefore help to both predict and plan for restoration and conservation strategies in this rapidly changing region.

## 2 Materials and Methods

### 2.1 Study System and Common Gardens

*Populus fremontii* populations are confined to riparian zones in the semi‐arid regions of the Southwestern USA, from California to New Mexico and Utah south into Mexico. In Arizona, populations are distributed along a north–south elevation gradient which correlates with mean annual temperature (MAT) and mean annual precipitation (MAP). *Populus fremontii* is a phreatophyte (Stromberg 1993) that is found in a range of hydrological conditions (Busch, Ingraham, and Smith 1992; Lite and Stromberg 2005; Smith et al. 1991; Snyder and Williams 2000), and population sites in this study have distinct climate and hydrological profiles (Table 1). Prior common garden studies involving *P. fremontii* have revealed patterns of both local adaptation and variation in phenotypic plasticity among genomically differentiated populations of this wide‐ranging tree species (Blasini et al. 2021, 2022; Cooper et al. 2019, 2022; Jeplawy et al. 2021).

**TABLE 1:** Source site (provenance) and common garden climate differences. CV VPD, coefficient of variation (CV) of annual VPD max; CV ΔVPD max, CV of annual change in VPD max; MAP, mean annual precipitation; MAT, mean annual temperature; VPD, maximum annual vapor pressure deficit. MAP and MAT from WorldClim (Fick and Hijmans 2017), VPD from PRISM (PRISM Climate Group, 2020), hydrology from USGS (National Hydrography Dataset Plus).

Site	Code	Elevation (m)	MAP (mm)	MAT (°C)	Mean VPD max (hPa)	CV VPD max (hPa)	CV ΔVPD max (hPa)	Hydrology
Populations								
Bill Williams, Colorado	LBW‐BIL	143	143	23.3	39.9	0.047	0.742	Perennial
San Luis, Colorado	SCT‐MEX	26	87	22.1	38.3	0.043	0.660	Perennial
San Pedro, Charleston	TSZ‐SAN	1219	320	17.4	29.5	0.053	0.996	Perennial
Agua Fria, Horseshoe	CAF‐AUG	988	384	17.3	28.7	0.056	0.710	Intermittent
Jack Rabbit, Little CO	JLA‐JAK	1507	213	12.6	24.5	0.065	0.788	Intermittent
Keams Canyon	KKH‐OPI	1920	280	10.4	18.7	0.071	0.697	Intermittent
Gardens								
Yuma	Hot	49	93	22.8				
Agua Fria	Warm	988	440	17.2				

**FIGURE 2:**
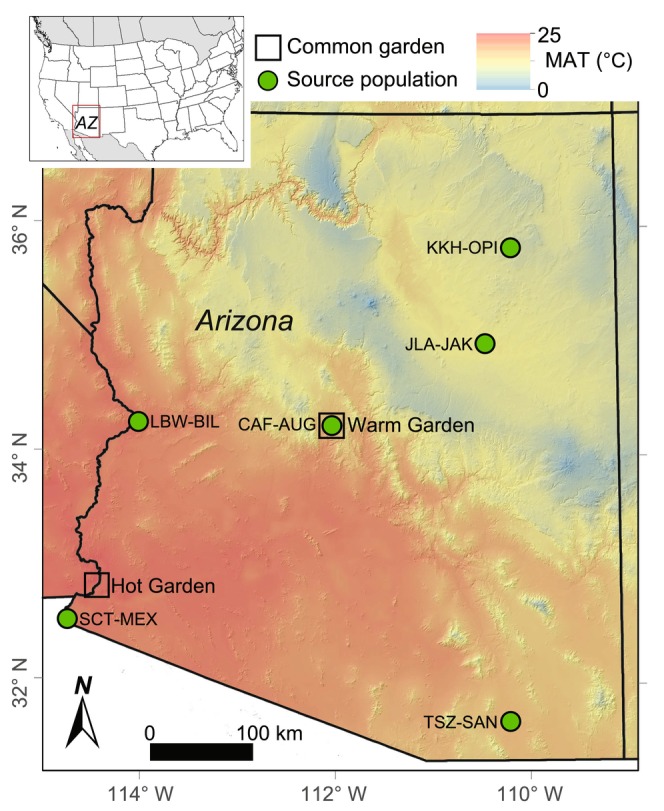
Study area including six provenance (source) sites and two common garden locations (squares) in the southwestern United States. Mean Annual Temperature (MAT) is lower in the northern region of the study area and 12.1°C higher in the southern region due to a steep elevation gradient. Additional provenance climate information is provided in Table 1.

Reciprocal common gardens along a climate gradient allow researchers to (a) test for evolved differences among populations while controlling environmental effects and (b) substitute space for time and quantify likely plant responses to future climate conditions. This study leverages two common gardens to quantify the phenotypic plasticity of leaf hydraulic architecture traits of *P. fremontii* . The common gardens used in this study were established in 2014 and have been previously described in detail (Cooper et al. 2019; Hultine et al. 2020a). The gardens span an elevational range from 49 to 988 m and a mean annual temperature (MAT) range of 17.2°C–22.8°C; (Table 1; Figure 2). We refer to the common gardens as “warm” (MAT = 17.2°C) and “hot” (MAT = 22.8°C) growing environments. The warm garden is near the center of this species' climatic range in central Arizona and the hot garden near Yuma, Arizona is near the extreme warm edge of this species' distribution. Together, these two gardens allow us to test how trees will respond to hotter growing conditions by tracking their plastic trait differences between warm average current temperatures and hot future temperatures. Optimal growth at these hot temperatures requires consistent access to water (Moran et al. 2023). Both common gardens were watered regularly from establishment through the course of this study (Cooper et al. 2019; Hultine et al. 2020a), but in periods of extreme heat, both likely experienced some water limitation. To establish the gardens, replicate cuttings were collected from multiple genotypes (i.e., individual wild trees) within each of 16 populations. All cuttings were rooted in the greenhouse for up to 4 months and planted at the common garden sites when saplings averaged 0.3 m in height. In this study, we focused on six populations from contrasting climates (Figure 2). These span 26–1920 m in elevation and 10.4°C–23.3°C in mean annual temperature (MAT; Table 1).

### 2.2 Leaf Collection

We assessed leaf hydraulic architecture for trees in each common garden. For each of the six populations, we randomly selected three target genotypes and three replicate trees per genotype (2 Environments (i.e., Gardens) × 6 Populations × 3 Genotypes × 3 replicates = 108 trees). For each tree, we collected 10 fully expanded leaves from the external southern side of the mid‐crown at ~1.5 m height between September and October of 2020, avoiding young leaves and those damaged by herbivory. Leaves were put on ice in the field and frozen in the lab. We randomly selected a single leaf of average size from each sample for each type of analysis (venation traits vs. stomatal traits). Some of these samples could not be obtained due to image quality during venation assessment, so that the final dataset includes *n* = 53 and *n* = 49 samples from the hot and warm gardens, respectively.

### 2.3 Leaf Hydraulic Architecture Traits

To assess venation traits on the collected leaves, we thawed leaf samples and cut 1 cm^2^ from the adaxial (upper) left side of the leaf between the primary vein and leaf margin (Figure 1). Leaf cuts focused on minor veins because they are largely independent of leaf size (Sack et al. 2012), which we know is separately affected by growing conditions in this species (Jeplawy et al. 2021). Leaf samples were cleared, stained, and mounted according to methods described by Pérez‐Harguindeguy et al. (2013) and Blonder and Enquist (2014). Image acquisition was performed using a Keyence digital microscope at 100× magnification; 9–16 images were taken of a single sample and stitched together to obtain a high‐resolution image (Figure 1a). We extracted leaf architecture features using LeafVeinCNN (Fricker, Blonder, and Xu 2020). LeafVeinCNN enables multiscale quantification of leaf vein networks using deep learning algorithms and convolutional neural networks (CNNs) (Xu et al. 2021). This automated software outperforms other network extraction methods and has a precision‐recall harmonic mean of 94.5% (Xu et al. 2021). We focused on four core venation traits including two traits related to how the veins were arranged within the leaf: Vein density (mm^1^ mm^−2^) and reticulation (mm^1^ mm^−2^), and two traits that quantify the dimensions of the leaf veins: Mean vein width (mm) and vein volume (mm^3^ mm^−2^) (Figure 1b).

For each leaf selected for stomatal analysis, a nail polish impression was taken from the adaxial (upper) surface on both sides of the midvein (the left and right sides of the leaf) (Hilu and Randall 1984). We did not assess abaxial stomata because a previous study on overlapping populations in one of the same common gardens found no variation in stomatal traits between adaxial and abaxial surfaces (Blasini et al. 2021). To take the impressions, clear nail polish was painted on both sides of the adaxial surface about mid‐leaf between two veins and from the mid‐vein to the edge of the leaf. When dry, the nail polish was peeled from the leaf and mounted on a microscope slide. Four images were taken at random locations on each slide under 100× magnification, and one image was taken at 400×. To assess density, we analyzed each 100× image for stomatal number using Stomata Counter (Fetter et al. 2019) which recognizes stomata via machine learning and then manually checked and adjusted each processed image. The four right‐side images were used to calculate a mean stomatal density (no. stomata mm^−2^) for each leaf sample. To assess size, we used ImageJ to manually measure the width and length of five closed stomata in each of the 400× images. We then combined density and size to calculate the theoretical maximum stomatal conductance (*g*_smax_) following Blasini et al. (2021) and Milla, de Diego‐Vico, and Martín‐Robles (2013).

### 2.4 Provenance Environmental Analysis

To characterize the climatic and hydrological conditions each population likely experienced over its recent evolutionary history (Table 1), we used three primary data sources. First, to compare average historical climate conditions among provenances, we obtained the earliest available 30 year normals (averages of annual data from 1960 to 1991) for mean annual temperature (MAT) and mean annual precipitation (MAP) using Climate WNA v4.62 (Hamann et al. 2013). Because temperature and precipitation in the Southwest are very strongly correlated with elevation, these recent climate variables are excellent proxies for the climates that trees have experienced during their local evolutionary histories, with *r* > 0.985 for correlations between current MAT and MAP (WorldClim 2, Fick and Hijmans 2017) and those variables estimated from 6000 or 22,000 years ago (WorldClim 1.4, Hijmans et al. 2005).

Second, because the evolution of plasticity may depend more on historical climate variation than historical averages (Botero et al. 2015; Leung et al. 2020), we also characterized interannual variation over a 30‐year period. Here, we focused on VPD max (maximum annual vapor pressure deficit), a key climate variable that integrates the effects of temperature and moisture on atmospheric drought (Hammond et al. 2022) by measuring the difference between the saturation vapor pressure and the actual vapor pressure (Daly, Smith, and Olson 2015). For plants, high VPD leads to greater evaporative demand at the height of the summer growing season due to either hotter temperatures, drier conditions, or both. This higher evaporative demand puts more stress on leaf veins moving water and stomata, balancing evaporative loss with photosynthesis and cooling. We calculated the mean and coefficient of variation (CV) of annual VPD max for each population provenance from 1981 to 2010 (obtained from PRISM Climate Group, 2020). To characterize interannual predictability in this variation, we also calculated the CV of the magnitude of change in VPD (delta VPD max) from 1 year to the next, obtaining a measure of each location's consistency in annual change. This allowed us to test whether higher and/or more predictable variation in a key climate variable could predict the evolution of trait plasticity across a species range. Finally, because selection on water use strategies in a riparian tree may depend not only on precipitation but also on ground and surface water availability, we used classifications from the USGS National Hydrography Dataset Plus version 2.1 (Brakebill, Schwarz, and Wieczorek 2020) to determine the stream hydrology (intermittent or perennial) at each population source site.

### 2.5 Tree Growth Measurements

To test whether leaf traits or their plasticity are related to whole tree performance, we assessed growth for each of our sampled trees over the 2020 growing season in each growing environment. We measured growth as the change in diameter at root crown (DRC, measured at 10 cm above the ground surface) between January 2020 and January 2021 (before and after the sixth growing season). For trees with multiple basal stems, we measured the diameter of each stem, calculated the basal area of each stem, summed these together, and then converted back to diameter. This serves to convert a multistemmed tree to a single stem with equivalent basal area, so that the increase in basal area can be calculated.

### 2.6 Statistical Analysis

To assess the magnitude of genetic (*G*), environmental (*E*), and *G* × *E* effects on leaf anatomical traits, we used linear mixed models (LMMs) implemented in the *lme4* package (Bates et al. 2015) of R (R Core Team 2022). We coded Environment (common garden, *n* = 2), Population (*n* = 6), and Population × Environment as fixed effects. We included Genotype and Genotype × Environment as random effects to capture genetic variation within populations. Since populations are genetically distinct, both Genotype × Environment and Population × Environment are levels of genetic × environment interactions (i.e., plasticity). From a statistical perspective, plasticity is a significant effect of environment on traits for the same genotype (a significant *G* × *E* effect in an LMM). For each trait response variable (vein density, vein reticulation, vein width, vein volume, stomatal density, stomatal size, and maximum stomatal conductance), we ran two different models to capture different subsets of genetic and environmental effects: (1) Full genetic × environment contrast: We used LMMs to understand the variation in leaf hydraulic architecture traits among and within all populations in our hot and warm environments. (2) Genetic effects in the hottest climate: We used LMMs to disentangle among and within population effects on vein traits in the hottest growing environment. This is important because hotter and drier environmental conditions are likely to become more prevalent over the range of *P. fremontii* in the future.

For each model, we checked model assumptions of homogeneity of variance and normality of residuals and random effects. We then assessed significance of the fixed effects using Type II and III Wald *F* tests with Kenward–Roger approximations for the denominator degrees of freedom, implemented using the ANOVA function in the *car* package (Fox and Weisburg 2019). Type III tests were used for models with significant interaction effects. Random effects were tested using likelihood ratio tests implemented in the *lmerTest* package (Kuznetsova, Brockhoff, and Christensen 2017). To compare the relative effects of environmental and genetic factors (Genotype & Population), we computed the variance explained by the fixed and random factors. Within the fixed effects, we compared the relative explanatory power of each effect using the partial R2 approach with the r2glmm R package (Jaeger 2017).

To test whether population differences in plasticity correspond to their differences in climate, we first calculated plasticity scores as the difference in genotype means across the hot and warm environments for each trait. We then used linear models (LMs) to test whether climate or hydrologic variables (Table 1) could predict the magnitude of plasticity for each genotype. We included population in these models to account for non‐independence among multiple genotypes from the same population. To compare the magnitude of plasticity among the traits in our study, we standardized each plasticity score by the maximum for that trait (Valladares, Sanchez‐Gomez, and Zavala 2006; (maximum mean‐minimum mean)/maximum mean).

Finally, we tested whether *P. fremontii* leaf hydraulic architecture traits and/or trait plasticity can predict plant performance. First, we tested whether vein or stomatal traits could predict tree growth in the hottest growing environment. We included the trait and population as fixed effects in an LMM and genotype as a random effect. Second, we tested whether the plasticity in vein or stomatal density for each genotype could predict average genotype growth across the hot and warm environments (Stinchcombe, Dorn, and Schmitt 2004; Van Kleunen and Fischer 2005). This regression tests the adaptive value of trait plasticity in this tree species by determining whether higher plasticity is associated with higher average growth rates. We used genotype plasticity score and population as predictor variables in an LM, with tree growth as the response variable.

## 3 Results

### 3.1 Environmental and Genetic Effects on Leaf Hydraulic Architecture Traits

**FIGURE 3:**
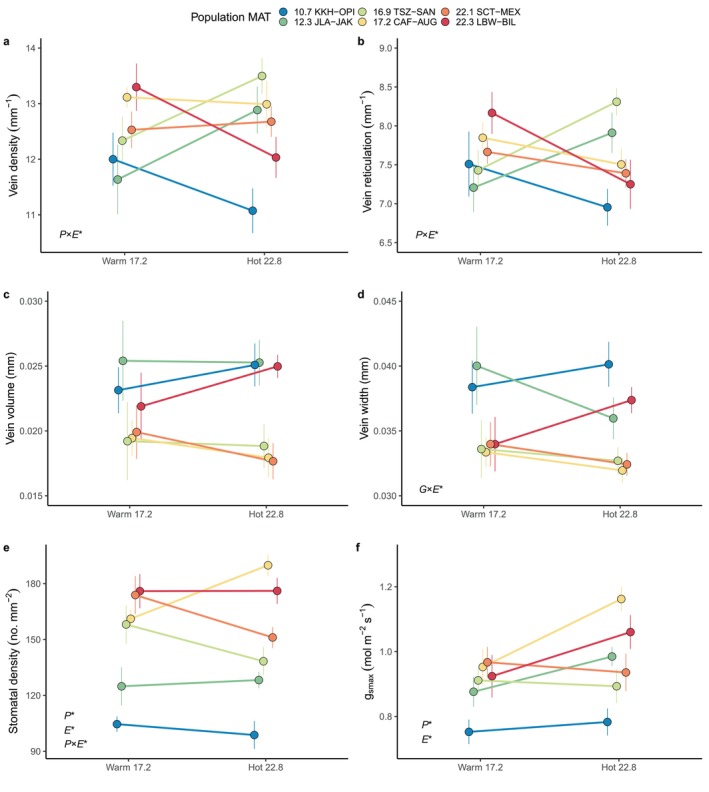
Reaction norms vary among populations (colors) for six traits (a–f) responding to two different common garden environments (Warm garden at 17.2°C Mean Annual Temperature and Hot garden at 22.8°C MAT). Points are means for each of the six populations and error bars are ±1 SE. Populations are colored by increasing MAT (Mean Annual Temperature in°C). *P* × E* = significant population × environment interaction; *G* × E* = significant genotype × environment interaction (see Table 2).

**FIGURE 4:**
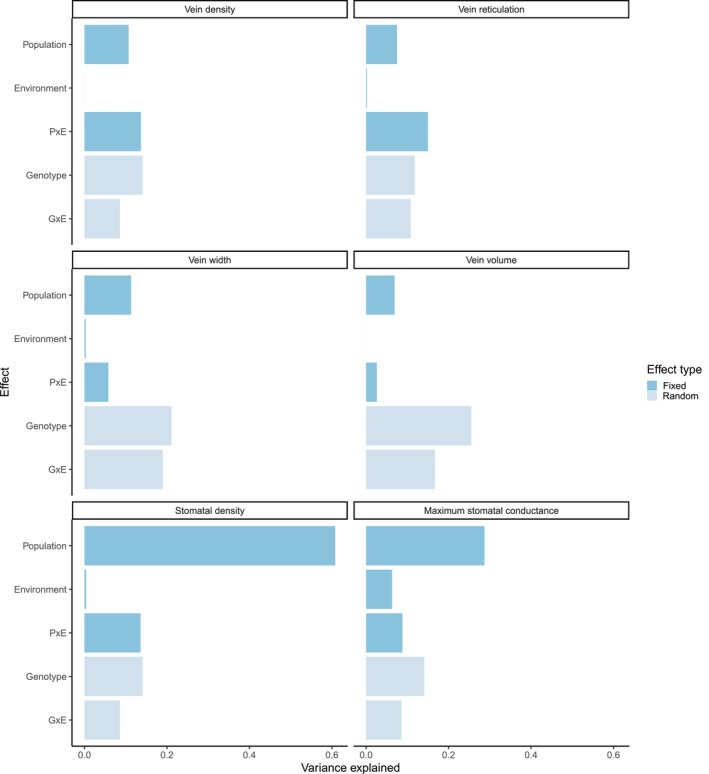
Variance explained by each effect in the full model testing for trait differences among populations (*P*, fixed) and common gardens (*E*, fixed) and genotypes (*G*, random) within populations. Statistical significance for these effects is given in Table 2.

As hypothesized, we found that *P. fremontii* leaf hydraulic architecture traits are phenotypically plastic across growing environments and that this plasticity varies among populations and, in some cases, genotypes. Genetic × environment interactions were particularly important for venation, where not only the magnitude but also the direction of plasticity varied widely (Figure 3). Leaves in hotter environments can benefit from higher vein density to increase water transport, but surprisingly not all genotypes responded in this way. Vein arrangement traits (vein density and reticulation) were mostly clearly shaped by Population × Environment effects, while vein dimension traits (width and volume, negatively related to arrangement traits) varied at an even finer genetic level and were better explained by random Genotype and Genotype × Environment effects within populations (Figure 4, Table 2). Stomatal density was also partly shaped by a Population × Environment effect (Table 2) but was primarily explained by Population (Figure 4). Finally, theoretical maximum stomatal conductance, the joint result of stomatal density and size, differed by population and environment (Figure 4).

**TABLE 2:** Environmental, genetic, and *G* × *E* effects on leaf hydraulic architecture traits, from linear mixed models. Significant effects (with *p* < 0.05) are in bold. These effects are plotted in Figure 3 with relative variance explained in Figure 4.

		Vein density			Vein reticulation			Vein width			Vein volume		
		df	*F*	*p*‐Value	df	*F*	*p*‐Value	df	*F*	*p*‐Value	df	*F*	*p*‐Value
Warm and hot environments													
Environment (Env)	Fixed	1, 10.81	0.05	0.831	1, 10.86	0.80	0.391	1, 11.22	0.26	0.620	1, 11.90	0.00	0.990
Population (Pop)	Fixed	5, 21.95	1.58	0.208	1, 22.83	1.09	0.391	5, 21.07	1.43	0.256	5, 11.98	1.53	0.253
Pop × Env	Fixed	5, 11.86	3.12	**0.050**	5, 11.86	3.25	**0.045**	5, 11.95	1.07	0.424	5, 11.96	0.50	0.771
Genotype (Geno)	Random			0.216			0.336			0.151			0.096
Geno × Env	Random			0.356			0.295			**0.035**			0.060
Hot environment													
Pop	Fixed	5, 11.90	2.68	0.076	5, 11.85	2.50	0.090	5, 11.93	3.00	0.056	5, 11.92	2.90	0.061
Geno	Random			**0.021**			0.071			**0.013**			**0.016**

		Stomatal density			Stomatal size			*g*_smax_		
		df	*F*	*p*‐Value	df	*F*	*P*‐Value	df	*F*	*p*‐Value
Warm and hot environments										
Environment (Env)	Fixed	1, 11.57	7.54	**0.018**	1, 11.78	7.13	**0.021**	1, 11.83	7.27	**0.020**
Population (Pop)	Fixed	5, 24.51	15.08	**< 0.001**	5, 11.94	16.90	**< 0.001**	5, 11.82	7.39	**0.002**
Pop × Env	Fixed	5, 11.89	3.12	**0.049**	5, 11.83	0.45	0.804	5, 11.88	1.96	0.158
Genotype (Geno)	Random			1			0.101			0.783
Geno × Env	Random			1			1			1
Hot environment										
Pop	Fixed	5, 11.92	23.79	**< 0.001**	5, 11.95	11.95	**< 0.001**	5, 11.91	7.57	**0.002**
Geno	Random			0.836			0.507			0.836

**FIGURE 5:**
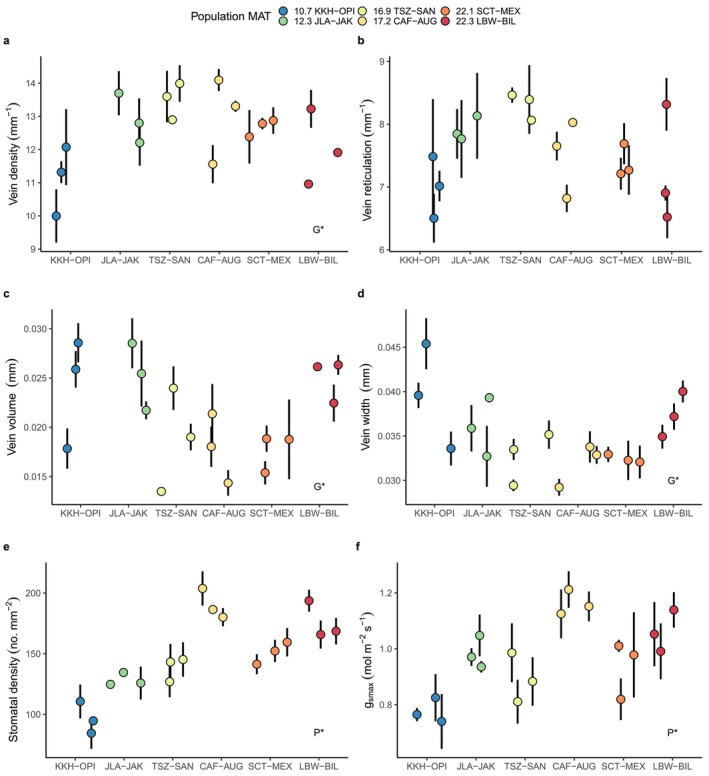
Leaf architecture traits differ across both populations and genotypes when grown in the hottest garden. Colored points are means for each genotype, error bars are ±1 SE. Populations are colored and ordered from left to right by increasing MAT (Mean Annual Temperature in°C). G*, significant genotype effect; P*, significant population effect.

These differences in the determinants of venation vs. stomatal traits are also clearly visible within the hottest growing environment (Figure 5). Under these conditions, leaf venation traits were explained by both Population (~28%–37% of variation) and Genotype (~22%–28% of variation), but only genotype effects were significant due to the lower degrees of freedom needed to model random effects (Table 2). In contrast, stomatal density, stomatal size, and theoretical maximum stomatal conductance were much more clearly differentiated among Populations (Table 2, Figure 4). Furthermore, populations from hot conditions did tend to have higher densities of small stomata, as expected (Figure 5e).

### 3.2 Independent Plasticity in Venation and Stomata

For our second hypothesis we predicted that trees would show coordinated strategies for water use that linked venation and stomatal traits, but this was not supported. As suggested by the differences in factors determining these two sets of traits, changes in one did not consistently predict changes in the other. For example, the two coldest populations shifted their vein density in opposite directions from the warm to hot garden (Figure 3a), but neither adjusted their stomatal density (Figure 3e) or stomatal size (not shown). This indicates that the multitrait constraints on hydraulic architecture are substantially different among populations and growing environments.

### 3.3 Predicting the Magnitude of Plasticity From Provenance Climate

**FIGURE 6:**
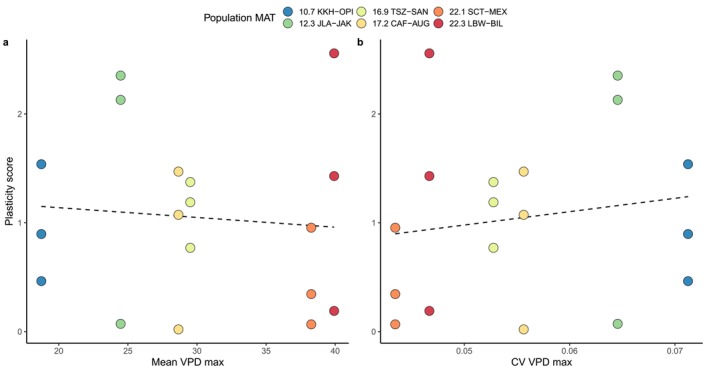
Provenance environment does not predict the magnitude of vein density plasticity across genotypes for all climate variables analyzed (mean VPD max, CV VPD max, not shown: MAP, MAT, delta CV VPD max, stream type and PC1). Dashed lines indicate non‐significant relationships (for all cases *p* > 0.45 for slope). Populations are colored by increasing MAT (Mean Annual Temperature in°C).

**FIGURE A1:**
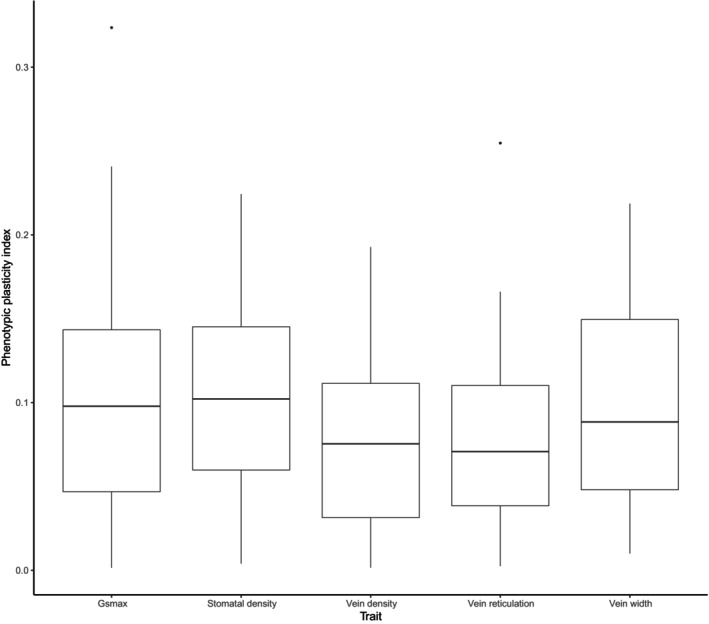
Comparison of phenotypic plasticity index values ((max value − min value)/max value) for five leaf hydraulic traits.

Contrary to our third hypothesis, plasticity in vein density was not predictable from population source climate (Figure 6). Neither measures of average climate conditions (MAT, MAP, and Mean VPD max) nor measures of variability (CV VPD max, CV of annual change in VPD max, and intermittency of stream flow) explained population variation in vein density plasticity (Table A1). This was also true when we used a multivariate PCA axis combining 21 climate variables (Cooper et al. 2019). Part of the reason for this lack of connection between population climate and phenotypic plasticity is that vein density plasticity scores varied widely among genotypes from the same population, with some populations showing a wider range of plasticity scores than others. However, all traits in our study showed similar levels of plasticity between the hot and warm environments (Figure A1), but no traits were consistently predictable from source climates. Even stomatal density, which was clearly differentiated among populations, showed similarly low plasticity in the hottest and coldest populations and divergent plasticity in climatically adjacent populations (Figure 3).

### 3.4 Consequences of Traits and Trait Plasticity for Performance

**FIGURE 7:**
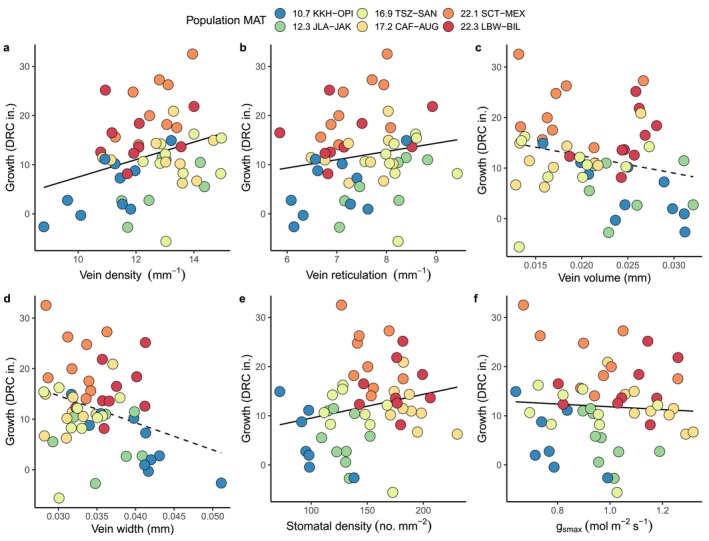
Leaf hydraulic architecture trait values predict tree growth in the hottest growing environment. Points are values for each tree; solid lines indicate significant effects of traits on growth (*p* < 0.05, see Table A2). Populations are colored by increasing MAT (Mean Annual Temperature in°C).

In partial agreement with our final hypothesis that traits of local populations would predict their higher growth, we found that higher vein density, vein reticulation, and stomatal density were associated with higher tree growth rates in the hottest environment (Figure 7). This is not surprising given the clear benefit of higher water conductance ability in hot and arid climates. Also, as expected, populations had significantly different growth in the hot environment (*p* = 0.001), with those from hot provenances growing more than those from colder provenances (Figure 7). However, higher theoretical maximum stomatal conductance had a weak negative effect on growth, which was unexpected (Figure 7). Also, contrary to our hypothesis, higher genotype plasticity in vein or stomatal density did not predict higher average genotype growth across the warm and hot environments (vein density: *F* = 0.002, *p* = 0.967; stomatal density: *F* = 1.43 *p* = 0.257). Thus, the ability to adjust trait values (i.e., plasticity) across these two environments was not beneficial for above‐ground productivity.

## 4 Discussion

Increasing temperature stress is likely to drastically change the environments to which *P. fremontii* populations are locally adapted (Cooper et al. 2019; Ikeda et al. 2017; Moran et al. 2023; Walsh et al. 2014). Phenotypic plasticity in leaf traits related to water use and gas exchange may constitute an important adaptive strategy that enables foundation tree species to persist under these changing environmental conditions (Nicotra et al. 2010). As predicted, our common garden study demonstrates that populations and genotypes differ in both their mean leaf hydraulic architecture in a single environment and their trait plasticity across environments. Furthermore, almost all of the leaf venation and stomatal traits we measured were clearly associated with tree growth under hot conditions, meaning that population mean differences are likely adaptive. Across growing environments, however, genotypes adjusted their traits in ways that decoupled links between veins and stomata and failed to maximize their growth across these conditions. For example, hotter populations produced higher densities of smaller stomata consistently in all environments, whereas their relative vein densities showed high plasticity across environments (Figure 3). In general, plasticity in leaf venation varied in both magnitude and direction even among populations from similar climates. Thus, the amount of plasticity across populations was difficult to predict, despite having major consequences for tree performance. These complex interactions between genetic identity and environmental conditions mean that leaf hydraulic conductance, water use efficiency, and thermal tolerance may all depend on which populations and genotypes are present as well as the magnitude of future environmental change.

### 4.1 Both the Magnitude and Direction of Plasticity Have Diverged Among Populations

Our study revealed a complex mosaic of phenotypic plasticity across the range of *P. fremontii* that is not simply driven by a steep linear gradient in climate (Figure 3). For vein density, we observed three types of responses among populations moving from the warm to hot environments. First, two of the populations in our study (hot SCT‐MEX and moderate CAF‐AUG) showed only minor variation in their vein traits across the warm and hot growing environments (Figure 3). These populations may be relying on stable mean trait values that help them perform sufficiently in both environments, as has been suggested for other tree species (Valladares et al. 2005). Second, two populations from opposite ends of the *P. fremontii* range (hot LBW‐BIL and cold KKH‐OPI) both decreased leaf vein density from the warm to the hot environment, whereas the other cold population (JLA‐JAK) and a moderate climate population (TSZ‐SAN) both increased vein density. Thus, populations from similar climates did not show similar plasticity.

Surprisingly, plasticity in vein traits was also not related to plasticity in stomatal traits. Stomatal density showed increasing, decreasing, or flat responses from warm to hot gardens, but an individual population's response for vein density did not predict its response for stomatal density (Figure 3). Interestingly, clear genetic × environmental interactions in stomatal density also did not translate into *G* × *E* effects on theoretical maximum stomatal conductance because plasticity in stomatal size was much more similar across populations. Taken together, this shows that even closely connected traits defining leaf hydraulic architecture can respond to environmental change in very different ways. This is surprising because adjustments in vein dimensions and architecture and stomatal density and size should all affect fitness in trees trying to maximize water use efficiency in hot conditions (Goodrich, Waring, and Kolb 2016; Sack and Scoffoni 2013). Previous work has shown that vein density and stomatal density are often coordinated (Brodribb, McAdam, and Carins Murphy 2017; Zhao et al. 2017). However, some studies have also found decoupled variation in leaf venation and stomatal traits and suggested this may evolve under heterogeneous environmental conditions (Pereira et al. 2022; Zhang et al. 2012). Heterogeneity among populations in the strength of temperature vs. water stress as selective forces could help explain our finding of independent differences in the means and plasticities of these vein and stomatal traits. Alternatively, it may be that some traits contribute more to fitness than others or that some are more easily modified to match environmental conditions. Additionally, vein traits may have been shaped by selection from herbivores damaging venation networks (Read and Stokes 2006) as well as climate, whereas stomatal traits appear more clearly related to provenance climate (Figure 5).

### 4.2 Why Is Plasticity So Unpredictable?

Because plasticity varied dramatically among populations from similar provenances, we found no evidence for increased plasticity in more climatically stressful or variable environments (Figure 6). This is in contrast with some previous studies on *P. fremontii* showing relationships between source site climate and phenological and phytochemical plasticity (Cooper et al. 2019; Eisenring et al. 2022). Vein and stomatal traits are often correlated with climate variables (Blonder and Enquist 2014; Bourne et al. 2017; Hetherington and Woodward 2003; Liu et al. 2018; Uhl and Mosbrugger 1999), but not always (Wang et al. 2019). We explored two possible reasons for the lack of predictability in these traits. First, it may be that the macroscale climate information available for each provenance does not completely capture the local hydraulic stresses driving tree growth and trait expression. This may be because more microscale climate data are needed (Maclean and Early 2023) and/or because different variables are needed beyond temperature, precipitation, and their combined effects on vapor pressure deficit. For example, the riparian tree species in our study may also depend on local stream hydrology and below‐ground access to water in addition to climate. Although we did not find any differences in plasticity between ephemeral and permanent streams, local hydrological records of groundwater depth at the specific population locations on those rivers over time could improve the power of this test.

For a tree species that is confined to riparian ecosystems with localized hydrological patterns (Butler Jr. et al. 2007; Snyder and Williams 2000), leaf hydraulic architecture trait plasticity may have evolved in response to selection pressures related to variability in both climate and groundwater. Thus, intra‐ and inter‐annual groundwater depth should be integrated with precipitation to account for the water gradient that this species can occupy. Mature *P. fremontii* trees growing along ephemeral streams can increase their water uptake from shallow soil layers by 33% after a summer precipitation event (Snyder and Williams 2000). However, this capacity may be reduced in the southern hot end of the range where the evaporative demand is too great and connection to groundwater becomes more important (Blasini et al. 2021; Hultine et al. 2020b). Across this range of conditions, phreatrophytic species may also use phenotypic plasticity of root traits (Guevara et al. 2010) as well as shoot and leaf traits (Pan et al. 2006) to cope with variation in groundwater availability. We did not find a simple effect of river ephemerality on plasticity in leaf traits, but future research could focus on a more complete characterization of annual and seasonal variation in water limitation across provenances, as well as integrating trait responses from roots to leaves (Sniderhan, McNickle, and Baltzer 2018).

The second possible reason that phenotypic plasticity in these traits is not predictable from population climates is that there is substantial genotypic variation in plasticity within populations. Multiple *Populus* species have demonstrated high intraspecific variation in vein density (Blonder, Violle, and Enquist 2013) and stomatal density (Blasini et al. 2021). Here, we found that populations varied widely in the direction of plasticity in some of these traits, but the magnitude of plasticity alone often varied more among genotypes within populations. For example, we found that the magnitude of plasticity for vein density and stomatal density was not significantly different among populations (Table A1; Population *p* > 0.05) due to genotype‐level variation. Allowing for variation in both magnitude and direction of plasticity uncovered significant population differences within environments, but this variation was still not predictable from provenance climates. Thus, unlike for other *Populus* traits (Cooper et al. 2019; Eisenring et al. 2022), much of the variation in the plasticity in hydraulic architecture cannot be explained by climatic differences across the landscape. Instead, we may need to understand finer‐scale data on variation in selective environments within populations, or other mechanisms that maintain local variation in genotype plasticity (i.e., herbivory and competition).

### 4.3 Consequences of Leaf Hydraulic Architecture Plasticity

Tree populations or genotypes with high levels of plasticity are likely to persist under future climate warming scenarios if the plasticity is adaptive, meaning that it provides a fitness benefit (Ghalambor et al. 2007). In this study, reaction norms differed among populations but we could not determine underlying drivers or consequences of this variation. Vein and stomatal density predicted growth in the hottest growing environment, but genotype plasticity scores for these two traits were not related to average growth across the warm and hot environments. There are a few possible reasons for this observation. Models to test effects of plasticity on average growth across conditions could be improved by using weighted growth values that account for the frequency that each environmental condition is encountered at each provenance, if those data are available (Van Kleunen and Fischer 2005). Alternatively, leaf anatomical plasticity may also be supporting functions in the plant other than aboveground growth such as leaf thermoregulation which may prevent damage to photosynthetic processes during heat events at the expense of reduced carbon uptake (Aparecido et al. 2020; Blasini et al. 2022). Third, plasticity in leaf hydraulic architecture and theoretical maximum stomatal conductance do not necessarily correspond directly to plasticity in a functional outcome like leaf turgor loss point or osmotic potential. Previous work in aspen, a congener of cottonwood, found high plasticity in some hydrological traits but limited drought response in others (Kerr et al. 2022). Additional research on the combined effect of genetic and plastic components of many component traits in a functional system will help to determine how growth and survival are achieved across temporal and spatial climatic variation.

### 4.4 Leaf Hydraulic Architecture Traits Predict Plant Performance

Although plasticity across environments did not predict growth across environments, we did find that vein arrangement traits and stomatal density were positively related to growth at hotter temperatures (Figure 7). Interestingly, these traits were not associated with growth at the more moderate temperatures in the warm environment. This result suggests that high vein arrangement and high stomatal density may be under selection in the extreme hot edge of the *P. fremontii* range. Because leaf veins are metabolically costly for plants to produce (Sack and Scoffoni 2013), the benefits of investments in dense and redundant network geometry will only outweigh the costs in environments where this network is necessary to avoid damage from factors such as extreme temperatures, drought, or herbivory. High vein density and stomatal density (such as we found in many of the populations from warmer provenances, Figure 5) may support leaf hydraulic functioning during periods of low soil water availability, high temperatures, and high aridity (Hetherington and Woodward 2003; Roth‐Nebelsick 2001). Under warm conditions high vein density and stomatal density supports a higher theoretical maximum stomatal conductance which is critical for leaf cooling under well‐watered conditions. Likewise, a high vein density could help isolate xylem embolism when leaf water potentials drop to a critical threshold and high stomatal density could facilitate rapid stomatal responses to changes in leaf water potential (Franks and Beerling 2009). These environmental parameters are closely associated with southern edges of the *P. fremontii* range. Climates that more closely resemble the warm and hot environments in our study are likely to expand by 38% by 2050 (Ikeda et al. 2017). The *P. fremontii* populations that can shift their leaf hydraulic architecture traits toward a phenotypic optimum for higher aridity are more likely to perform better in these warming climates.

### 4.5 *Populus fremontii* Populations in a Changing Environment

Plasticity is known to vary within and among plant species but has been explored for a limited number of traits in riparian trees and is rarely examined across a species' full climatic range. This knowledge gap is important because studying plasticity across a species' range can allow us to test its role in both past local adaptation and performance under predicted environmental conditions. Results from this study suggest that different *P. fremontii* populations will vary in their capacity to adjust their leaf hydraulic architecture and support growth in a warmer environment. Populations adapted to the colder Utah High Plateau are predicted to face a 99% reduction in suitable habitat by 2050 (Ikeda et al. 2017). As warming continues, populations in this region may show contrasting or limited plastic responses in leaf hydraulic architecture traits and subsequently experience very different vulnerabilities to thermal stress. Phenotypic plasticity in leaf hydraulic architecture traits, if adaptive, may promote survival for populations that are likely to suffer rapid environmental change during their lifetimes. Restoration practitioners could apply these results (Figure 3, Table 2) and use plastic genotypes such as JLA‐JAK (a cold population) or genotypes with beneficial vein trait values such as SCT‐MEX (a hot population) in future restoration projects that will occur in an increasingly arid Southwest (Walsh et al. 2014). Research that continues to quantify phenotypic plasticity, determine above‐ and below‐ground environmental drivers of that plasticity, and test for the adaptive value of plasticity will improve predictions of plant responses to changing environments.

## Author Contributions

**Iris J. Garthwaite:** conceptualization (lead), data curation (lead), formal analysis (lead), investigation (lead), methodology (lead), writing – original draft (lead), writing – review and editing (lead). **Catherine Lepp:** data curation (equal), formal analysis (equal). **Zyled S. R. Maldonado:** data curation (equal), formal analysis (equal). **Davis Blasini:** methodology (equal). **Kevin C. Grady:** funding acquisition (equal), resources (equal), writing – review and editing (equal). **Catherine A. Gehring:** funding acquisition (equal), resources (equal), writing – review and editing (equal). **Kevin R. Hultine:** funding acquisition (equal), resources (equal), writing – review and editing (equal). **Thomas G. Whitham:** funding acquisition (equal), resources (equal), writing – review and editing (equal). **Gerard J. Allan:** funding acquisition (equal), resources (equal), writing – review and editing (equal). **Rebecca J. Best:** conceptualization (equal), data curation (equal), formal analysis (equal), funding acquisition (lead), investigation (equal), methodology (equal), project administration (equal), resources (equal), supervision (equal), writing – original draft (equal), writing – review and editing (equal).

## Conflicts of Interest

The authors declare no conflicts of interest.

## Data Availability

Data are available in the Dryad repository: https://doi.org/10.5061/dryad.zcrjdfnpb.

## Appendix 1

**TABLE A1:** Predictability of genotype plasticity scores for vein density from environmental conditions at the population provenances (results from a linear model for each climate variable). Stomatal density plasticity scores also ns for all environmental variables. delta VPD max (cv), CV of annual change in VPD max; MAP, mean annual precipitation; MAT, mean annual temperature; PC1, PCA axis combining 21 climate variables; stream type, intermittent or perennial; VPD max (cv), coefficient of variation (CV) of annual VPD max; VPD, maximum annual vapor pressure deficit. PC1 from Cooper et al. 2019, MAP and MAT from WorldClim (Fick and Hijmans 2017), VPD from PRISM (PRISM Climate Group, 2020), hydrology from USGS (National Hydrography Dataset Plus).

	df	*F*	*p*‐Value
PC1	1, 12	0.449	0.516
Population	4, 12	0.691	0.612
VPD max (mean)	1, 12	0.114	0.741
Population	4, 12	0.774	0.563
VPD max (cv)	1, 12	0.368	0.556
Population	4, 12	0.711	0.600
Delta VPD max (cv)	1, 12	0.548	0.474
Population	4, 12	0.666	0.628
Stream type	1, 12	0.105	0.752
Population	4, 12	0.777	0.561
MAT	1, 12	0.323	0.580
Population	4, 12	0.722	0.593
MAP	1, 12	0.002	0.963
Population	4, 12	0.802	0.547

**TABLE A2:** Relationships between leaf hydraulic architecture traits and growth in the hot environment (results from a linear mixed model for each trait). Significant effects (with *p*  < 0.05) are in bold.

		df	*F*	*p*‐Value
Vein density	Fixed	1, 40.02	7.242	**0.010**
Population	Fixed	5, 11.94	9.247	**0.001**
Geno	Random			0.604
Vein reticulation	Fixed	1, 41.95	7.709	**0.008**
Population	Fixed	5, 11.72	11.226	**< 0.001**
Geno	Random			0.697
Vein width	Fixed	1, 36.15	0.967	0.332
Population	Fixed	5, 11.93	8.742	**0.001**
Geno	Random			1.000
Vein volume	Fixed	1, 36.16	0.008	0.930
Population	Fixed	5, 11.60	9.183	**0.001**
Geno	Random			1.000
Stomatal density	Fixed	1, 44.92	8.694	**0.005**
Population	Fixed	1, 13.52	11.972	**< 0.001**
Geno	Random			0.710
*g*_smax_	Fixed	1, 44.56	7.037	**0.011**
Population	Fixed	5, 12.91	13.781	**< 0.001**
Geno	Random			1.000
